# Patient Privacy in the Era of Big Data

**DOI:** 10.4274/balkanmedj.2017.0966

**Published:** 2018-01-20

**Authors:** Mehmet Kayaalp

**Affiliations:** 1 National Library of Medicine, National Institutes of Health, Maryland, ABD

**Keywords:** Health Insurance Portability and Accountability Act, medical informatics, confidentiality, data anonymization data sharing, personally identifiable information, privacy

## Abstract

Privacy was defined as a fundamental human right in the Universal Declaration of Human Rights at the 1948 United Nations General Assembly. However, there is still no consensus on what constitutes privacy. In this review, we look at the evolution of privacy as a concept from the era of Hippocrates to the era of social media and big data. To appreciate the modern measures of patient privacy protection and correctly interpret the current regulatory framework in the United States, we need to analyze and understand the concepts of individually identifiable information, individually identifiable health information, protected health information, and de-identification. The Privacy Rule of the Health Insurance Portability and Accountability Act defines the regulatory framework and casts a balance between protective measures and access to health information for secondary (scientific) use. The rule defines the conditions when health information is protected by law and how protected health information can be de-identified for secondary use. With the advents of artificial intelligence and computational linguistics, computational text de-identification algorithms produce de-identified results nearly as well as those produced by human experts, but much faster, more consistently and basically for free. Modern clinical text de-identification systems now pave the road to big data and enable scientists to access de-identified clinical information while firmly protecting patient privacy. However, clinical text de-identification is not a perfect process. In order to maximize the protection of patient privacy and to free clinical and scientific information from the confines of electronic healthcare systems, all stakeholders, including patients, health institutions and institutional review boards, scientists and the scientific communities, as well as regulatory and law enforcement agencies must collaborate closely. On the one hand, public health laws and privacy regulations define rules and responsibilities such as requesting and granting only the amount of health information that is necessary for the scientific study. On the other hand, developers of de-identification systems provide guidelines to use different modes of operations to maximize the effectiveness of their tools and the success of de-identification. Institutions with clinical repositories need to follow these rules and guidelines closely to successfully protect patient privacy. To open the gates of big data to scientific communities, healthcare institutions need to be supported in their de-identification and data sharing efforts by the public, scientific communities, and local, state, and federal legislators and government agencies.

## Evolution of privacy

Privacy and confidentiality have been two of the major pillars of medical ethics since Classical Antiquity, albeit they may not always have been expressed in those terms (2). The definition and the extent of privacy have been a constant struggle for scholars and philosophers, which is still true today (3,4). In the old days, it simply implied secrets of a person. In the early modern period, with the concept of *my home is my castle*, privacy enveloped the personal space. The Attorney General of England Sir Edward Coke stated it in 1604 as “The house of every one is to him as his castle and fortress, as well for his defence against injury and violence as for his repose” (5). This idea found it’s home in the U.S. Constitution with the Fourth Amendment in 1791 as “The right of the people to be secure in their persons, houses, papers, and effects, against unreasonable searches and seizures,...” (6).

In 1890, Warren and Brandeis (7) defined privacy as “the right to be let alone,” which is still in use by various contemporary authors (8,9,10). In 1948, United Nations General Assembly adopted the Universal Declaration of Human Rights and enshrined privacy as a fundamental human right: “No one shall be subjected to arbitrary interference with his privacy, family, home or correspondence, nor to attacks upon his honour and reputation. Everyone has the right to the protection of the law against such interference or attacks” (11). However, there was still no consensus on what constituted privacy or on its extent or limits (3,4,12,13,14,15,16).

As our lives get more complex, the concept has become more involved and complicated. Some defined privacy in terms of solitude (7,17) or control accessibility to oneself (3), anonymity (4), autonomy (2,18,19) or control over one’s own body and sexuality (3,4). With the advent of digital communication revolution, as social media becomes ubiquitous, the concept of privacy has evolved organically. Today, we define it as *the right to maintain control over personal information* (20), which is information about oneself, including information about one’s possessions, communications, conducts, and other affairs. Physical intrusion into the personal space or obstruction of personal conducts may co-occur with the invasion of privacy but physical aspects of such breaches are defined within the realms of other civil liberties; whereas, the right to privacy provides us the legal and ethical authority to determine how and with whom our personal information can be shared.

Despite being a fundamental human right, privacy is neither absolute nor unconditional; it is limited by the rights of others and by civic duties. For example, no person can choose not to disclose their income information from the government revenue services on the grounds of privacy, but the government agency cannot disclose such personal information to others without the person’s permission or without a court order. Although income tax is held as private information in the US, this is not true everywhere in the world (21) as the understanding of what is private, what needs to be disclosed to the public, and where lies the boundary between public interest and privacy differ among cultures.

## Protection of health information containing patient identifiers

In the United States, any biomedical study that includes personal identifiers of an individual who is the subject of the information is categorized as human subjects research (22,23). Health information (HI) is defined as information related to the past, present, and future health care or health status of the individual or information related to health care payments. Individually identifiable health information is a *subset* of HI (24) and contains identifiers or other such information that can be used to identify the subject of the health information (25). Most of the individually identifiable health information are protected health information (PHI) the main exceptions are those found in education records (e.g., immunization records) not maintained by a healthcare provider (26), in employment records, and health information of individuals deceased more than 50 years ago. Health information of the individual deceased within the last 50 years is considered PHI (25).

Individually identifiable information, also known as personally identifying information (PII), is frequently confused with individually identifiable health information. Some PII elements such as personal names and social security numbers can be found in medical records, but they are not health information, hence not PHI. We consider PHI as the set at the intersection of health information and PII (Figure 1a, b, c). Although this particular set representation of PHI is true, it can be misinterpreted as PII in health records unless we elaborate on constituents of these sets and their relations.

In Figure 1a, *C* is the set of all elementary (noncompound) clinically pertinent information and *PID* is the set of all elementary personal identifiers. These two sets overlap because some *PID* elements (e.g., age, gender, and ethnicity) entail clinically pertinent information. Since the set of elementary information at the intersection of *C* and *PID* is mostly demographic in nature, it is labeled as *D*, but *D* also contains some non-demographic PII such as medical record numbers. All other elements of *PID* such as personal names and telephone numbers comprise the set *P*. The set of health information *H *consists of all clinically pertinent information elements that are not in *D* (e.g., “has Parkinson’s disease”).

Elements of the sets *H, D*, and *P* in Figure 1a are denoted with the corresponding lower case letters and distinguished from each other with distinct suffixes (Figure 1b). Any single health record such as *R_1_* in Figure 1a, b, c may include a particular combination of these information elements. If a record such as *R_2_* in Figure 1a is a subset of *H* only, it would comprise health information not linked to an individual; thus, it would not constitute PHI (see the tan area in Figure 1c). If a document such as *R_3_* in Figure 1a contains *PID* elements with no elements of *H* (e.g., a table of names and addresses), it would not be considered as health information (see the pink area of Figure 1c). Note that each element of the sets in Figure 1a is elementary information (e.g., first name = “John”, age = “35”), whereas elements of the sets in Figure 1c are compound information (i.e., any combination of elementary information); hence, a record is a member of those sets in Figure 1c. A health record is PHI, only if it contains elements from both *H* and *PID* (see the orange area in Figure 1c). Note that some seemingly elementary information such as “hospital admission date” may be a set of compound information [e.g., {*h_i_, d_j_*} = {“admitted to the hospital”, “on June 1, 2017”}].

The U.S. Public Health law supports federally funded studies on sensitive health issues such as sexual attitudes, sexually transmitted diseases, addictions, mental health, and illegal behaviors, by protecting PHI of those research subjects. Researchers of such studies may apply to the National Institutes of Health (NIH) to receive Certificates of Confidentiality (27). Researchers with certificates of confidentiality can disclose neither PHI nor PII of the research subjects collected for that particular study to “any Federal, State, or local civil, criminal, administrative, legislative, or other proceeding” even after the federal funding is concluded. The disclosure of PHI by researchers would only be allowed under certain conditions that have been specifically permitted by the human subject in his/her informed consent (28).

## HIPAA Privacy Rule

In 1996, the U.S. Congress enacted the Health Insurance Portability and Accountability Act (HIPAA) and required the Secretary of Health and Human Services (HHS) to promulgate standards to address. “(1) The rights that an individual who is a subject of individually identifiable HI should have. (2) The procedures that should be established for the exercise of such rights. (3) The uses and disclosures of such information that should be authorized or required” (29). In 1999, HHS proposed the initial version of the Privacy Rule as a set of privacy protection standards for handling and transmitting HI of individuals (30). The current version of the rule incorporated amendments of the Health Information Technology for Economic and Clinical Health (HITECH) Act and the Genetic Information Nondiscrimination (GINA) Act of 2008 (23).

The Privacy Rule prohibits selling PHI (31) or using it for marketing purposes unless a written authorization is obtained from the individual for that specific purpose. If the remuneration is received by the selling/marketing party, it must be explicitly stated (32).

PHI can be used by the provider for the purpose of the individual’s care (i.e., for primary use) and disclosed to other providers for the same purpose or disclosed to health insurance services for payment notifications. PHI can also be disclosed to another person (e.g., a family member) designated by the individual. The individual has the right to be informed prior to any such disclosure and to restrict those disclosures. Upon the death of the individual, the provider can disclose PHI to a family member or designated person unless the individual had a request against such disclosures (33).

PHI can also be used or disclosed for secondary (non-care related) use in limited circumstances. If legally required, the provider may disclose PHI without the individual’s authorization to a public health or government authority, to authorized programs related to workers’ compensation, or to organizations involved in tissue/organ banking or transplantations (34).

Disclosures must always be limited to the minimum PHI that is “necessary to accomplish the intended purpose of the use, disclosure, or request” (31). Organizations can allow employee access only to those pieces of PHI that are appropriate and necessary to perform their duties (35). For example, a hospital registrar may access patients’ names and addresses but not their diagnostic codes or clinical reports.

## Privacy rule for research

Researchers can use PHI if they receive authorizations from the subjects of PHI. Without such authorization, also called informed consent, (36,37,38) researchers must apply to the institutional review board (IRB) for a waiver of authorization (22). The minimum necessary PHI can be released to researchers without prior authorization from the individual upon the approval of a waiver of authorization by an IRB or a privacy board (31,34,35).

Healthcare institutes can grant researchers access to PHI without an IRB approval in two limited cases: (a) The data can be disclosed to researchers if PHI belong to deceased individuals, (b) If the request is limited to reviewing PHI for a preparatory study to research and no PHI leaves the institute (34).

Waivers of authorization can be provided by IRBs only if the requested PHI is necessary for the study, the proposed study is deemed scientifically sound and important, the protocol is well planned, and all necessary safety, security and privacy measures are taken. Furthermore, researchers must demonstrate that;

1. “The research involves no more than minimal risk to the subjects.

2. The waiver or alteration will not adversely affect the rights and welfare of the subjects.

3. The research could not practicably be carried out without the waiver or alteration, and

4. Whenever appropriate, the subjects will be provided with additional pertinent information after

participation” (36).

Unless researchers substantiate why all PII elements preserved in the PHI are necessary, PII elements must be removed from the data prior to disclosing it to the study (31). The process of removing PII elements from PHI is known as de-identification.

## De-identification

**Figure 2:**
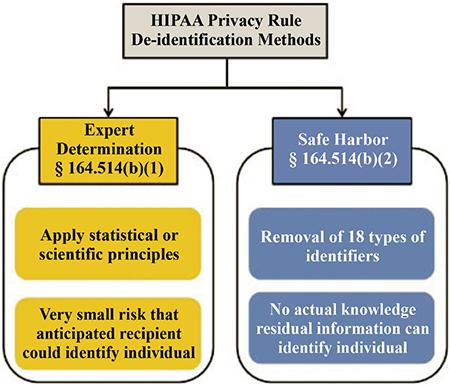
Graphical representation of HIPAA Privacy Rule de-identification methods. *Source: Office of Civil Rights, Department of Health and Human Services (39)*. *HIPAA: Health Insurance Portability and Accountability Ac*

The Privacy Rule provides two different de-identification methods (Figure 2) (23,39). In the first method, expert determination, an expert de-identifies PHI, documents the methodology, and quantifies the minimal risk of re-identification using generally accepted statistical and scientific methods.

The second one called the Safe Harbor method requires 18 types of identifiers (PII elements) to be removed from data (Table 1). If the de-identifying party is aware of other PII elements that remained in the de-identified data and could identify the individual, they should be removed as well.

Demographic information most frequently used in clinical studies such as age, gender, ethnic origin and occupation are not included in the set of 18 PII elements. The exception is the category of ages 90 and above, which the Privacy Rule requires to be combined into a single age category, because in a given location, the size of the population at this particular age can be so small that the age information by itself can be used to identify the individual.

The Privacy Rule establishes a specific provision called Limited Data Set (LDS) when one or more of the 18 PII elements are required for a study. Since LDS would contain PII elements, the resulting HI is considered PHI, but institutes are allowed to disclose LDS to researchers without IRB review if researchers sign a data use agreement established by the institute.

If researchers can demonstrate that their study requires the following PII elements for research purposes, they can obtain this information within an LDS:

1.All date elements (i.e., no longer limited to year information, incl. birth date) and age information (incl. 90 and above),

2. Full ZIP code, town, city, and county information.

Expert Determination method is usually offered by commercial de-identification services with the claim that the Safe Harbor method removes necessary demographic and date information (40). Such claims hold no water given that within the framework of LDS, researchers can access all such information without an IRB review if and when they can demonstrate that such information is necessary for the study.

## De-identification of different data types

There are four types of data: tabular, image/video, signal, and text data. De-identifying tabularly structured data is straightforward if specifications of the fields are defined. In most databases, records are represented in lines and fields in columns. If a particular column contains one of the 18 PII elements, we can easily redact the contents of that column for all records. Genomic data is tabular in nature.

Privacy Rule dictates that full-face photographic images and images that can identify the individual should be removed, but it is possible to remove only the facial characteristics from pictures. For example, the faces of all individuals presented in Google Street View images (41,42,43,44) are blurred as are the letters and numbers on license plates of motor vehicles. To accomplish this task with images, one needs to use face and text recognition applications (45,46).

DICOM is the most prevalent standard to represent and transmit clinical image data. Each DICOM image dataset comprises a header of structured (tabular) data and pixels of images. DICOM images can be de-identified mainly by de-identifying the tabular data section only; however, there are de-identification tools that recognize and de-identify “burned-in annotations” (46) or blur the facial characteristics on brain MR images, without any distortion of clinical image information of the brain (45).

Most signal data such as electrocardiography and electroencephalography do not require de-identification. The only signal data required to be de-identified is voiceprints, which are graphical representations of individual voices in terms of frequency, amplitude and duration. Through mathematical analysis, individuals can be re-identified from their voiceprints; thus, they are considered biometric identifiers like fingerprints.

Genomic data is also unique to every individual. It is possible to detect a particular genomic sequence (of an individual) in a large collection of genomic sequences (47). In this sense, the nature of genomic data is similar to fingerprints. Note, however, neither gene sequences nor fingerprints by themselves can identify the individual. To use fingerprints for identification, an external information source, mapping each particular fingerprint to a personal name and other identifiers of that person, must be available. The only successful method in the literature for the re-identification of individuals whose genomic data were stored in a public site was using not the genomic data but the birthdate, full ZIP code and gender information (48). By removing all unnecessary demographic, date and geographic information from genomic databases and minimizing essential demographic information (e.g., by aggregating age information), the risk of a privacy breach can be greatly reduced. If a breach does happen, it would unlikely occur during the study of genomic data, but rather via the disclosure of other information mapping the specimen number or the genomic data to standard identifiers (i.e., PII) of the individual.

Text data is a much larger component of the electronic health record (EHR) than tabular data (49,50,51,52). Clinical narratives can provide indispensable information to research studies about the patient’s clinical condition that other data types cannot (53). However, de-identifying free text data is more complicated than de-identifying tabular data because there is no schema similar to what databases have and no well-defined structure of records and fields. With no controlled vocabulary, expressions in English or in any other natural language are intrinsically vague such that the same word (e.g., “may”) can have multiple meanings in different contexts; thus, distinguishing health information from PII can be challenging.

Still it is feasible to manually de-identify clinical reports if the amount of text is limited and if experienced de-identification professionals are available and well trained. In the era of Big Data, the first premise is rarely applicable. Manual de-identification can be very expensive for institutions and would become infeasible as the number of studies relying on clinical reports increases. Data providers may have little or no incentive to perform manual de-identification. Although they may charge only a nominal fee for such a service, by law, providers cannot seek any profit by providing the data. To provide manual de-identification services, institutes would have to undertake huge burdens of operational and financial overhead as well as risk negative publicity if the process failed.

## Types of automatic text de-identification systems

Fortunately, there are automatic clinical text de-identification tools whose sensitivity and accuracy increase continuously. PARAT (54) and De-IDTM (55) are two such tools that are commercially available. There are also other freely available software applications such as MIST (56), de-id (57), and National Library of Medicine (NLM)-Scrubber (58,59,60) developed at MITRE, MIT and NIH, respectively. Both MIST and de-id have been academic exercises without a long-term plan for improvement and maintenance.

NLM-Scrubber was developed with a long-term goal of providing a non-commercial solution to biomedical scientists and institutions that do not have necessary resources to undertake the clinical text de-identification process. As a government research organization, NIH has no profit motive. The project aims to provide US taxpayers the best patient privacy protection and allows them to benefit from rapid scientific advances.

Current de-identification systems use various methods to recognize PII in clinical data, but neither a survey (61,62,63,64) of current de-identification systems nor computational techniques and mathematics of de-identification is within the scope of this review. However, clinical data scientists need to know what to expect from a de-identification system, how it can be used, and most critically, what types of input they need to provide the system to receive the desired output.

## Annotated health information

Some clinical text de-identification systems such as MIST use supervised machine learning (ML) methods and require a set of training data where each PII element is annotated manually. Such ML systems either come with an annotation tool or are capable of using outputs of existing annotation tools (65). Annotation is a precursor of de-identification performed by human experts (66); thus, an ML system learns from the annotations of human experts, and attempts to recognize PII elements in the non-training data and to replicate the human performance during the de-identification process. Other de-identification systems such as NLM-Scrubber operate without training data.

All health institutes that de-identify health records need to employ human experts to annotate a small subset of randomly selected health records from the data of the larger cohort that needs to be de-identified. This small set of annotated HI would serve as the gold standard to evaluate the performance of the automatic de-identification system of the institute (67,68). Without such an evaluation and verification, a health institute would not know if the output of the de-identification system is truly de-identified.

An ML system requires annotated training data that is usually much larger than the annotated gold standard data. Unfortunately annotated gold standard data cannot be reused for training purposes since the two need to be mutually exclusive; otherwise, the evaluation results would be misleading. The size of the training data depends on the learning ability of the system and on the complexity of the data that needs to be de-identified; thus, an institute has to train the system in iterative steps with increasing size of training data, until the size increase does not significantly improve the system’s performance. Due to the open-ended nature of the training data production and the large size of the prerequisite training data, the overhead of creating training datasets may be overwhelming for health institutes that lack the necessary human resources to carry out the task.

## Modes of de-identification

From the clinical data scientist point of view, an automatic clinical text de-identification application is a black box; that is, the application takes some input and produces de-identified data—the underlying mechanism of de-identification does not matter much as long as the application produces the desired output. To produce optimal results, the data scientist needs to know the various operation modes available for the de-identification system in hand.

An earlier study (69) distinguished eight modes of de-identification, to which we add a ninth, pseudonymization (Table 2). These modes define how the user can operate a given de-identification system if the system provides a particular functionality. The first three change the mode of operation in terms of de-identification time and input. The next two alter the input and output modes, respectively. The following three modes involve different stakeholders as active participants in the de-identification operation, and the last mode moves de-identification to the cloud. Most of these modes can be combined to maximize the protection of patient privacy and the integrity of the de-identified data.

*Repository-wide batch de-identification* is the default mode of operation adopted by most (if not all) existing systems. For an institute, it is tempting to de-identify its entire repository at once and make the de-identified data available to researchers when requested. In contrast, the next two modes de-identify data on demand. The repository-wide batch mode makes the data available at the time of request without additional operational overhead. However, the data might have been de-identified using an older technology with a lower quality of de-identification; the de-identified data may be incomplete and/or incorrect if the source data has been updated since the de-identification occurred; and it may not contain some of the required demographic information necessary for the study.

In *on-demand cohort-specific de-identification*, the data of the cohort that researchers defined is de-identified on demand. Since modern de-identification systems are very fast, the delay between the data collection and de-identification would be insignificant. *On-demand de-identification of query results* requires the integration of the de-identification system into the EHR system. Results of the query can be de-identified on the fly before being displayed to researchers.

By augmenting the input mode of de-identification with patient and provider identifiers, the accuracy of results can be improved significantly (58). In the pseudonymization mode, the de-identified data replaces PII elements (e.g., “Fred Jones”) with pseudonyms (e.g., “John Doe”) instead of with a label of the corresponding PII element (e.g., “[Personal Name]”), so if the system fails to de-identify some PII elements, the user might not be aware of the failure as remaining PII elements blend in among other pseudonyms (70).

In the *scientist involved de-identification* mode, scientists actively participate in the de-identification, producing better de-identification results. If the scientist’s active participation is ensured, the sensitivity of the de-identification system for recognizing PII elements can be increased manually. As a side effect of the increased sensitivity, some HI could be misidentified as PII. By reviewing the first batch of de-identified results, the scientist can identify a set of misidentified terms, which can then be input to the system, so those terms can be preserved during the second de-identification cycle. De-identification using this mode results in better protection of patient privacy and a more complete set of de-identified data with higher scientific value and data integrity.

*Patient involved de-identification* is hypothetical since no existing system currently offers patients to annotate their own records for de-identification purposes. In very rare occasions, the context of the narrative might inadvertently reveal the identity of the patient; e.g., “injured during his US championship match today” (71). In such cases, manual patient annotations would help improve de-identification results. Furthermore, as de-identified clinical reports become widely available to researchers, it is likely that patients would demand to be informed of which portions of their records are made available to researchers.

Physicians are occasionally required to cite the patient’s full name and medical record number to link the record to that specific patient but it is a generally unnecessary and unadvisable practice. It would be best if medical students are trained to write anonymous clinical reports without patient identifiers so that these reports can be used for scientific research purposes in the future. Using *physician involved de-identification* mode, the system warns physicians whenever they use patient identifiers. If such identifiers are necessary for clinical care purposes, they can be automatically labeled and those labels then verified by the physician.

As big health data becomes widely available to clinical scientists, it will likely be accumulated and accessed at large centers such as state cancer registries, state universities, and government research centers, which can allocate the expertise and necessary resources to handle big data and provide services to other institutes nationwide. The *online de-identification *mode would enable scientists of smaller institutes to access de-identified data of much larger cohorts. Centers holding big health data can act as honest brokers, de-identify the data, develop proper data use agreements, and monitor compliance of users.

## DISCUSSION

Protecting patient privacy requires various technical tools. It involves regulations for sharing, de-identifying, securely storing, transmitting and handling PHI. It involves privacy laws and legal agreements. It requires establishing rules for monitoring privacy leaks, determining actions when they occur, and handling de-identified clinical narrative reports. De-identification is one such indispensable instrument in this set of privacy tools.

Protecting patient privacy requires collaboration among all stakeholders, which include patients, PHI holding institutions, users of HI, developers of automatic de-identification tools, and regulatory and law enforcement government agencies. Each group has a different set of roles and responsibilities. For example, institutions should be held responsible to select the right tools, monitor the adequacy of these tools over time, and ensure the quality and content of de-identified data before presenting it to the user. They also are required to use these tools properly by supplying all necessary input to the de-identification system and utilizing all available modes of de-identification to maximize privacy protection. Institutions are also responsible to establish proper data use agreements.

Institutions and users of HI are equally responsible for ensuring that the requested and granted data comprise only the HI that is necessary for the study. Both regulatory agencies and institutions should empower patients to actively protect their privacy by monitoring their EHRs and let them know what portions of their data have been shared, with whom, and to what end. Institutions should demand from their users to provide study terms of interest to input to the de-identification process, so that the scientific integrity of the data can be preserved while privacy protection can be achieved at the highest level of sensitivity for de-identifying PHI.

As outlined above, the demand from institutions holding PHI is significant. Smaller institutions can be overwhelmed by the operational and financial overhead. There is little or no incentive structure for these institutions to take this challenge eagerly and share the data for secondary scientific use, particularly with scientists outside of those institutions. The entire scientific community including journal editors and the public, with the help of regulatory and grant providing agencies, should build incentive structures to support these institutions and make their contributions to the advancement of science visible.

In conclusion, Big Data makes the problem of patient privacy protection bigger and more difficult to attain; however, recent advances in computational de-identification help remedy the problem and enable scientists to access big health data by minimizing the risk to patient privacy. We have made great strides in developing both regulatory and technical privacy tools for the era of big data; however, this is still a work in progress.

We reviewed the progress of patient privacy protection with a focus on the U.S. As seen in references, regulations have been continuously updated with numerous amendments. We did not discuss the European efforts but the regulations there are more in flux. In 2016, the European Parliament enacted the General Data Protection Regulation (GDPR), which will take effect in 2018 (38). GDPR provides patient privacy protection using a language similar to the Privacy Rule.

Thanks to the digital communication revolution, the world gets smaller every day. As everyone deserves to equally benefit from scientific advances, it is inevitable that any legal differences among nations including U.S., Europe, Canada, and Australia will soon be ironed out so that we all can collaborate to find cures to today’s incurable diseases and improve the quality of life around the world.

## Figures and Tables

**Table 1:**
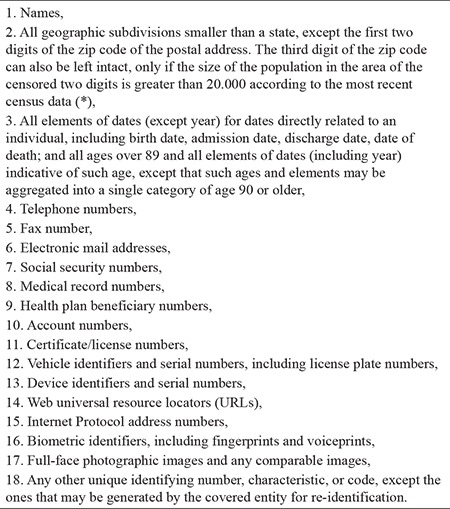
Per HIPAA Privacy Rule, the following identifiers must be removed from PHI to obtain fully de-identify health information (*). As of 2010, there were 18 sets of zip codes with distinct initial three digits whose corresponding population sizes were less than or equal to 20.000 (60)

**Table 2:**
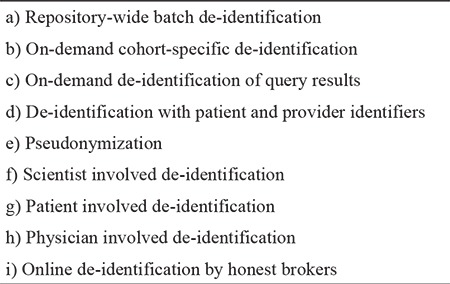
Modes of de-identification

**Figure 1a:**
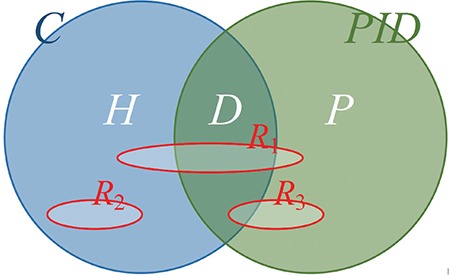
Relationship *between* the set C of all elementary clinical information and the set *PID* of all elementary personal identifiers, Their subsets are health information with no personal identifiers H, demographic information and clinical personal identifiers D, non-clinical personal identifiers *P*, and three hypothetical records R_1_, R_2_, and R_3_.

**Figure 1b:**
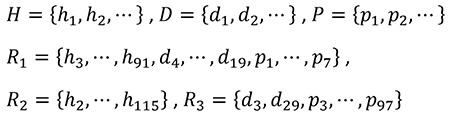
Relationship among *H, D*, and *P* and the representation of three hypothetical records using the set notation.

**Figure 1c:**
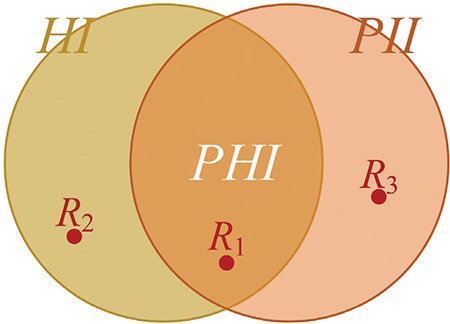
Protected health information *(PHI)* is the intersection of health information *(HI)* and personally identifying information *(PII)*. Members of all sets are compound information such as R_1_ a clinical report with personal identifiers, R_2_ a de-identified clinical report, and R_3_ a table of personal identifiers with no clinical connections.
